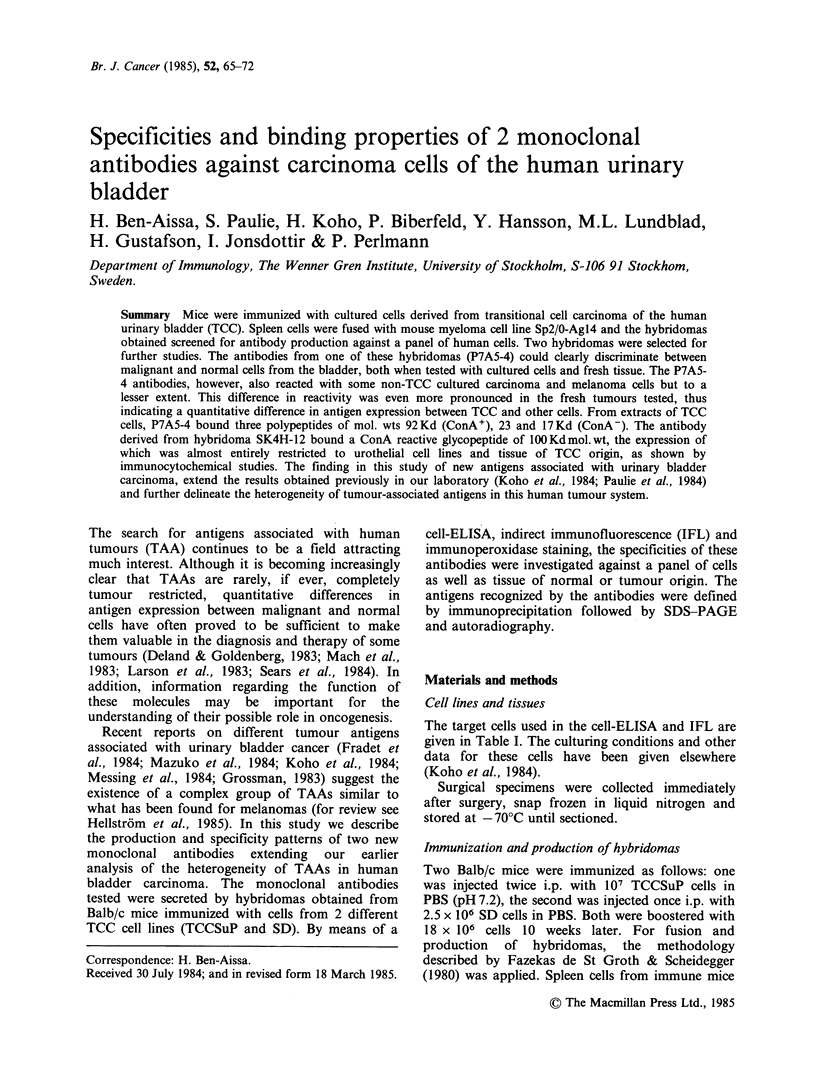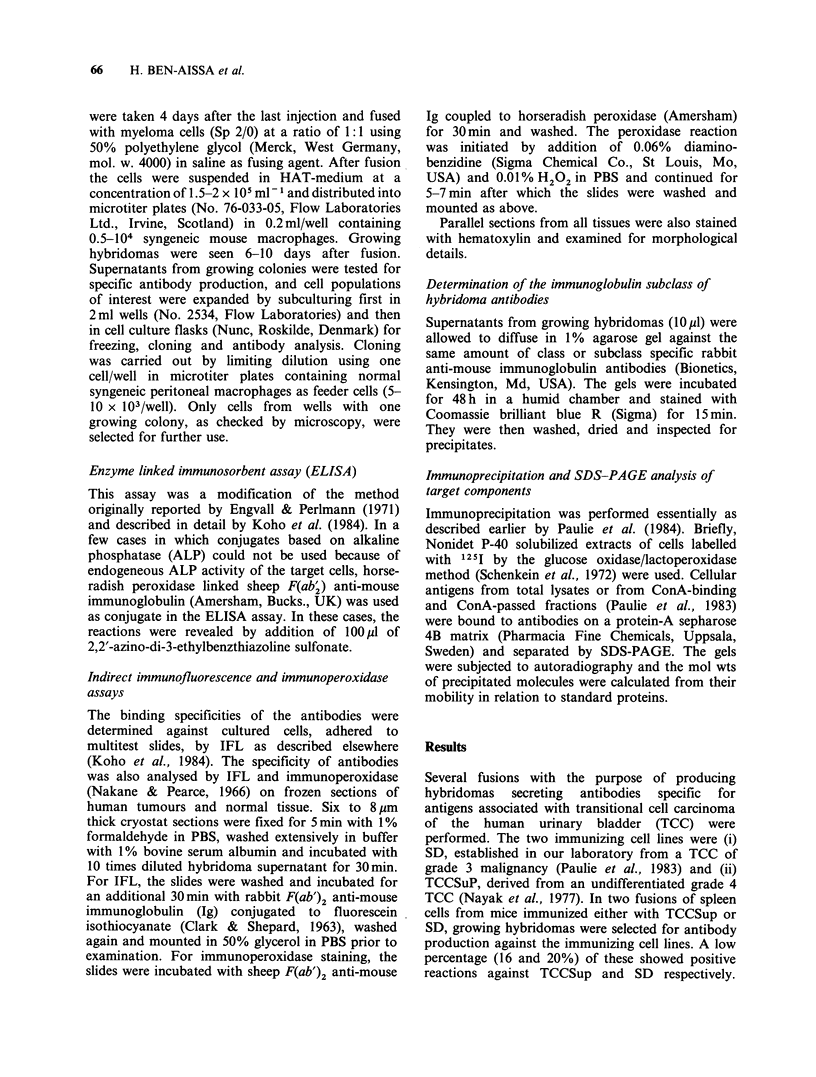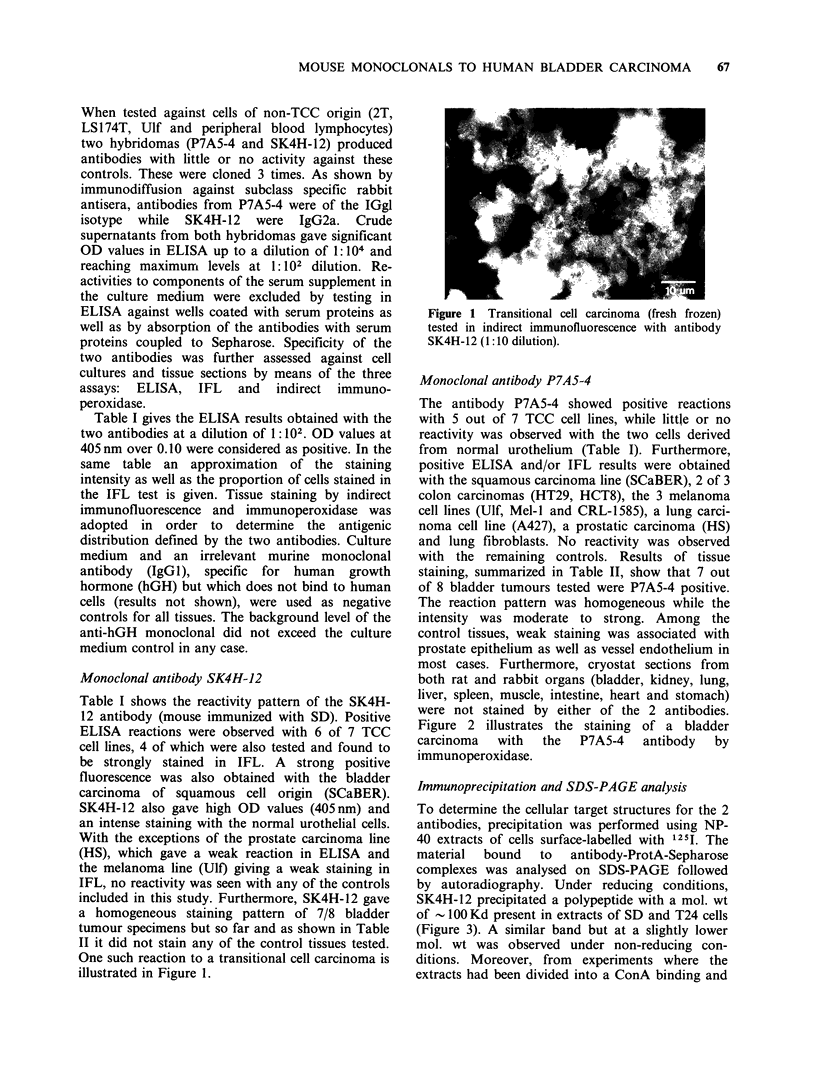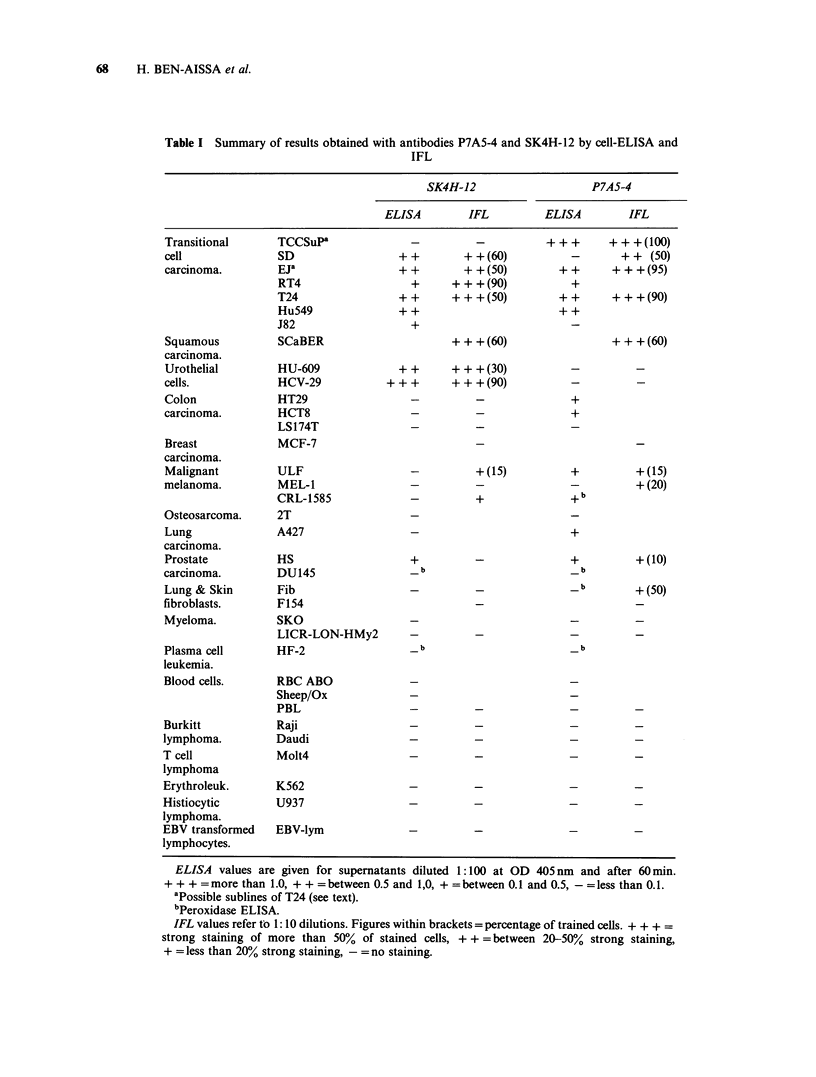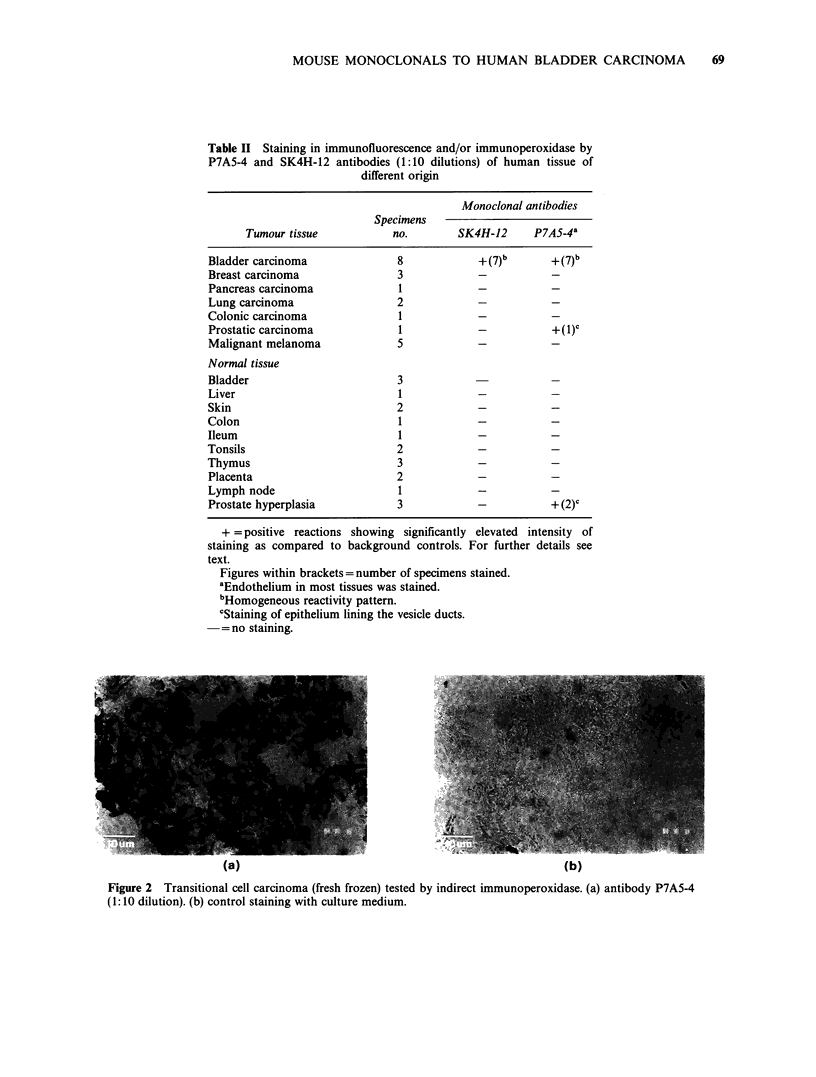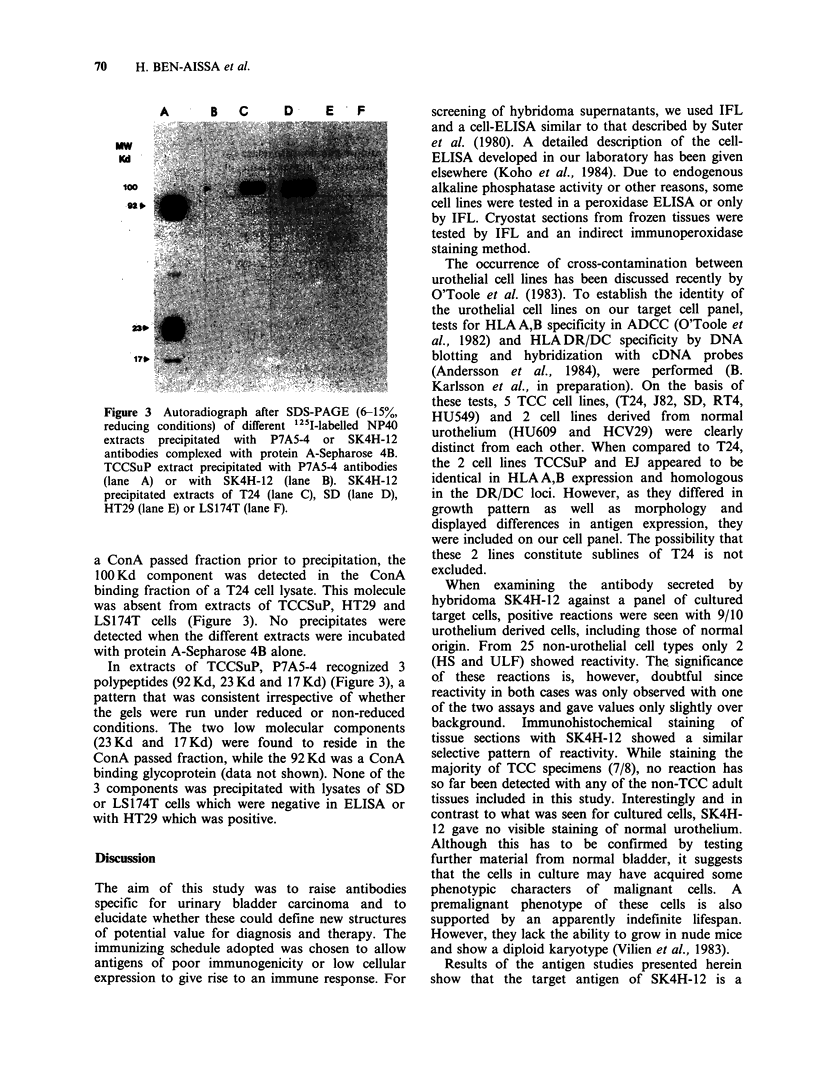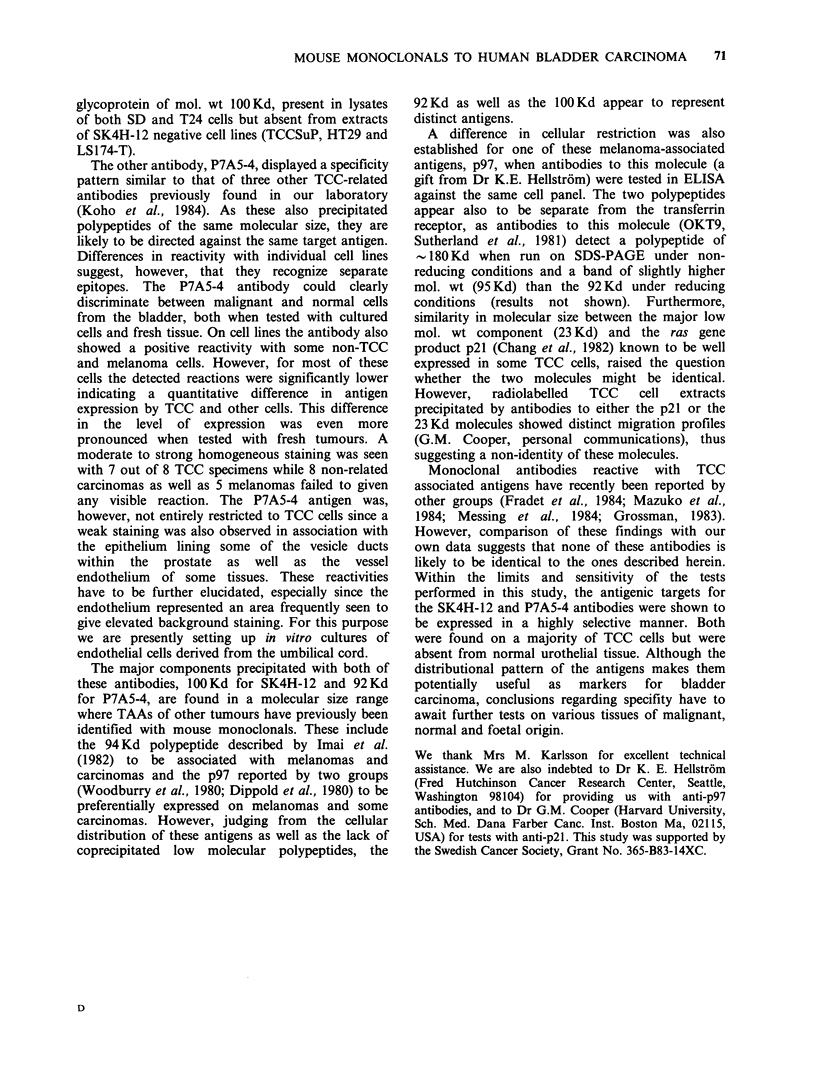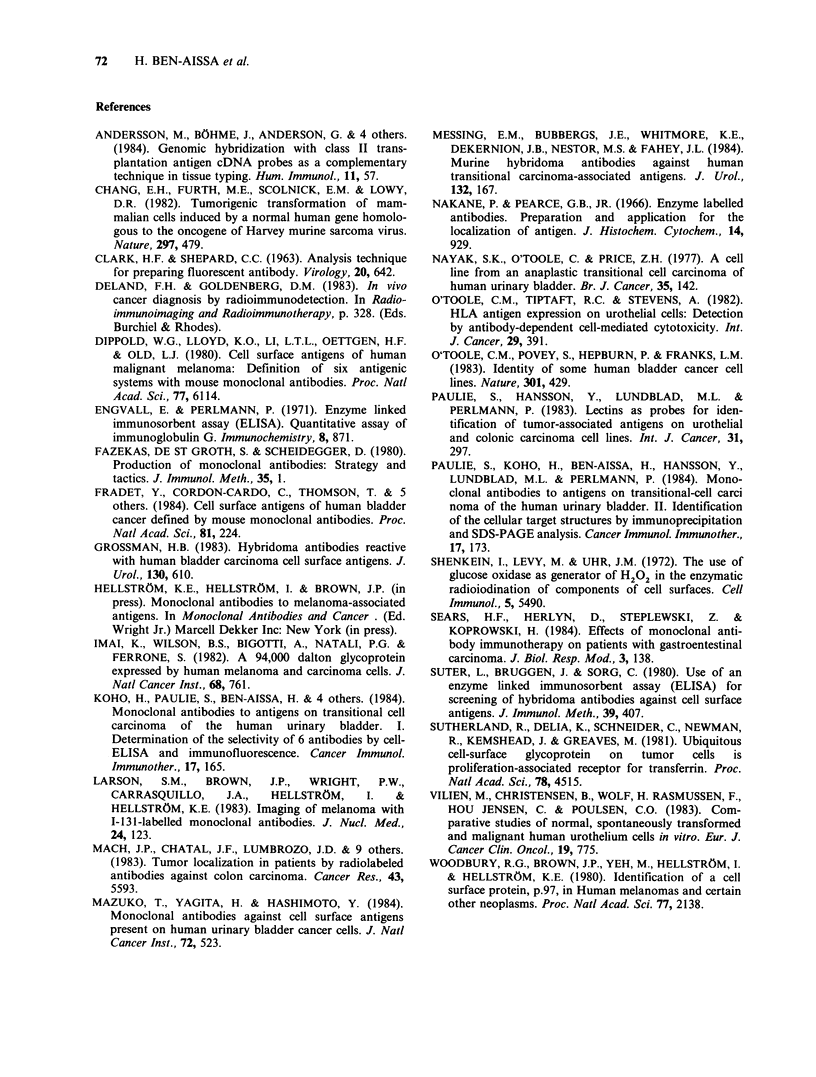# Specificities and binding properties of 2 monoclonal antibodies against carcinoma cells of the human urinary bladder.

**DOI:** 10.1038/bjc.1985.150

**Published:** 1985-07

**Authors:** H. Ben-Aissa, S. Paulie, H. Koho, P. Biberfeld, Y. Hansson, M. L. Lundblad, H. Gustafson, I. Jonsdottir, P. Perlmann

## Abstract

**Images:**


*Br. J. Cancer* (1985), **52**, 65–72

### Specificities and binding properties of 2 monoclonal antibodies against carcinoma cells of the human urinary bladder

H. Ben-Aissa, S. Paulie, H. Koho, P. Biberfeld, Y. Hansson, M.L. Lundblad, H. Gustafson, I. Jonsdottir & P. Perlmann

*Department of Immunology, The Wenner Gren Institute, University of Stockholm, S-106 91 Stockhom, Sweden.*

**Summary** Mice were immunized with cultured cells derived from transitional cell carcinoma of the human urinary bladder (TCC). Spleen cells were fused with mouse myeloma cell line Sp2/0-Ag14 and the hybridomas obtained screened for antibody production against a panel of human cells. Two hybridomas were selected for further studies. The antibodies from one of these hybridomas (P7A5-4) could clearly discriminate between malignant and normal cells from the bladder, both when tested with cultured cells and fresh tissue. The P7A5-4 antibodies, however, also reacted with some non-TCC cultured carcinoma and melanoma cells but to a lesser extent. This difference in reactivity was even more pronounced in the fresh tumours tested, thus indicating a quantitative difference in antigen expression between TCC and other cells. From extracts of TCC cells, P7A5-4 bound three polypeptides of mol. wts 92 Kd (ConA$^+$), 23 and 17 Kd (ConA$^-$). The antibody derived from hybridoma SK4H-12 bound a ConA reactive glycopeptide of 100 Kd mol. wt, the expression of which was almost entirely restricted to urothelial cell lines and tissue of TCC origin, as shown by immunocytochemical studies. The finding in this study of new antigens associated with urinary bladder carcinoma, extend the results obtained previously in our laboratory (Koho *et al.*, 1984; Paulie *et al.*, 1984) and further delineate the heterogeneity of tumour-associated antigens in this human tumour system.

The search for antigens associated with human tumours (TAA) continues to be a field attracting much interest. Although it is becoming increasingly clear that TAAs are rarely, if ever, completely tumour restricted, quantitative differences in antigen expression between malignant and normal cells have often proved to be sufficient to make them valuable in the diagnosis and therapy of some tumours (Deland & Goldenberg, 1983; Mach *et al.*, 1983; Larson *et al.*, 1983; Sears *et al.*, 1984). In addition, information regarding the function of these molecules may be important for the understanding of their possible role in oncogenesis.

Recent reports on different tumour antigens associated with urinary bladder cancer (Fradet *et al.*, 1984; Mazuko *et al.*, 1984; Koho *et al.*, 1984; Messing *et al.*, 1984; Grossman, 1983) suggest the existence of a complex group of TAAs similar to what has been found for melanomas (for review see Hellström *et al.*, 1985). In this study we describe the production and specificity patterns of two new monoclonal antibodies extending our earlier analysis of the heterogeneity of TAAs in human bladder carcinoma. The monoclonal antibodies tested were secreted by hybridomas obtained from Balb/c mice immunized with cells from 2 different TCC cell lines (TCCSuP and SD). By means of a cell-ELISA, indirect immunofluorescence (IFL) and immunoperoxidase staining, the specificities of these antibodies were investigated against a panel of cells as well as tissue of normal or tumour origin. The antigens recognized by the antibodies were defined by immunoprecipitation followed by SDS–PAGE and autoradiography.

Correspondence: H. Ben-Aissa.
Received 30 July 1984; and in revised form 18 March 1985.

### Materials and methods

### *Cell lines and tissues*

The target cells used in the cell-ELISA and IFL are given in Table I. The culturing conditions and other data for these cells have been given elsewhere (Koho *et al.*, 1984).

Surgical specimens were collected immediately after surgery, snap frozen in liquid nitrogen and stored at −70°C until sectioned.

### *Immunization and production of hybridomas*

Two Balb/c mice were immunized as follows: one was injected twice i.p. with $10^7$ TCCSuP cells in PBS (pH 7.2), the second was injected once i.p. with $2.5 \times 10^6$ SD cells in PBS. Both were boostered with $18 \times 10^6$ cells 10 weeks later. For fusion and production of hybridomas, the methodology described by Fazekas de St Groth & Scheidegger (1980) was applied. Spleen cells from immune mice

© The Macmillan Press Ltd., 1985

were taken 4 days after the last injection and fused with myeloma cells (Sp 2/0) at a ratio of 1:1 using 50% polyethylene glycol (Merck, West Germany, mol. w. 4000) in saline as fusing agent. After fusion the cells were suspended in HAT-medium at a concentration of $1.5–2 \times 10^5\ ml^{-1}$ and distributed into microtiter plates (No. 76-033-05, Flow Laboratories Ltd., Irvine, Scotland) in 0.2 ml/well containing $0.5–10^4$ syngeneic mouse macrophages. Growing hybridomas were seen 6–10 days after fusion. Supernatants from growing colonies were tested for specific antibody production, and cell populations of interest were expanded by subculturing first in 2 ml wells (No. 2534, Flow Laboratories) and then in cell culture flasks (Nunc, Roskilde, Denmark) for freezing, cloning and antibody analysis. Cloning was carried out by limiting dilution using one cell/well in microtiter plates containing normal syngeneic peritoneal macrophages as feeder cells (5–$10 \times 10^3$/well). Only cells from wells with one growing colony, as checked by microscopy, were selected for further use.

*Enzyme linked immunosorbent assay (ELISA)*

This assay was a modification of the method originally reported by Engvall & Perlmann (1971) and described in detail by Koho *et al.* (1984). In a few cases in which conjugates based on alkaline phosphatase (ALP) could not be used because of endogeneous ALP activity of the target cells, horse-radish peroxidase linked sheep $F(ab'_2)$ anti-mouse immunoglobulin (Amersham, Bucks., UK) was used as conjugate in the ELISA assay. In these cases, the reactions were revealed by addition of 100 $\mu$l of 2,2′-azino-di-3-ethylbenzthiazoline sulfonate.

*Indirect immunofluorescence and immunoperoxidase assays*

The binding specificities of the antibodies were determined against cultured cells, adhered to multitest slides, by IFL as described elsewhere (Koho *et al.*, 1984). The specificity of antibodies was also analysed by IFL and immunoperoxidase (Nakane & Pearce, 1966) on frozen sections of human tumours and normal tissue. Six to 8 $\mu$m thick cryostat sections were fixed for 5 min with 1% formaldehyde in PBS, washed extensively in buffer with 1% bovine serum albumin and incubated with 10 times diluted hybridoma supernatant for 30 min. For IFL, the slides were washed and incubated for an additional 30 min with rabbit $F(ab')_2$ anti-mouse immunoglobulin (Ig) conjugated to fluorescein isothiocyanate (Clark & Shepard, 1963), washed again and mounted in 50% glycerol in PBS prior to examination. For immunoperoxidase staining, the slides were incubated with sheep $F(ab')_2$ anti-mouse Ig coupled to horseradish peroxidase (Amersham) for 30 min and washed. The peroxidase reaction was initiated by addition of 0.06% diamino-benzidine (Sigma Chemical Co., St Louis, Mo, USA) and 0.01% $H_2O_2$ in PBS and continued for 5–7 min after which the slides were washed and mounted as above.

Parallel sections from all tissues were also stained with hematoxylin and examined for morphological details.

*Determination of the immunoglobulin subclass of hybridoma antibodies*

Supernatants from growing hybridomas (10 $\mu$l) were allowed to diffuse in 1% agarose gel against the same amount of class or subclass specific rabbit anti-mouse immunoglobulin antibodies (Bionetics, Kensington, Md, USA). The gels were incubated for 48 h in a humid chamber and stained with Coomassie brilliant blue R (Sigma) for 15 min. They were then washed, dried and inspected for precipitates.

*Immunoprecipitation and SDS–PAGE analysis of target components*

Immunoprecipitation was performed essentially as described earlier by Paulie *et al.* (1984). Briefly, Nonidet P-40 solubilized extracts of cells labelled with $^{125}I$ by the glucose oxidase/lactoperoxidase method (Schenkein *et al.*, 1972) were used. Cellular antigens from total lysates or from ConA-binding and ConA-passed fractions (Paulie *et al.*, 1983) were bound to antibodies on a protein-A sepharose 4B matrix (Pharmacia Fine Chemicals, Uppsala, Sweden) and separated by SDS-PAGE. The gels were subjected to autoradiography and the mol wts of precipitated molecules were calculated from their mobility in relation to standard proteins.

### Results

Several fusions with the purpose of producing hybridomas secreting antibodies specific for antigens associated with transitional cell carcinoma of the human urinary bladder (TCC) were performed. The two immunizing cell lines were (i) SD, established in our laboratory from a TCC of grade 3 malignancy (Paulie *et al.*, 1983) and (ii) TCCSuP, derived from an undifferentiated grade 4 TCC (Nayak *et al.*, 1977). In two fusions of spleen cells from mice immunized either with TCCSup or SD, growing hybridomas were selected for antibody production against the immunizing cell lines. A low percentage (16 and 20%) of these showed positive reactions against TCCSup and SD respectively.

When tested against cells of non-TCC origin (2T, LS174T, Ulf and peripheral blood lymphocytes) two hybridomas (P7A5-4 and SK4H-12) produced antibodies with little or no activity against these controls. These were cloned 3 times. As shown by immunodiffusion against subclass specific rabbit antisera, antibodies from P7A5-4 were of the IGgl isotype while SK4H-12 were IgG2a. Crude supernatants from both hybridomas gave significant OD values in ELISA up to a dilution of $1:10^4$ and reaching maximum levels at $1:10^2$ dilution. Reactivities to components of the serum supplement in the culture medium were excluded by testing in ELISA against wells coated with serum proteins as well as by absorption of the antibodies with serum proteins coupled to Sepharose. Specificity of the two antibodies was further assessed against cell cultures and tissue sections by means of the three assays: ELISA, IFL and indirect immunoperoxidase.

Table I gives the ELISA results obtained with the two antibodies at a dilution of $1:10^2$. OD values at 405 nm over 0.10 were considered as positive. In the same table an approximation of the staining intensity as well as the proportion of cells stained in the IFL test is given. Tissue staining by indirect immunofluorescence and immunoperoxidase was adopted in order to determine the antigenic distribution defined by the two antibodies. Culture medium and an irrelevant murine monoclonal antibody (IgG1), specific for human growth hormone (hGH) but which does not bind to human cells (results not shown), were used as negative controls for all tissues. The background level of the anti-hGH monoclonal did not exceed the culture medium control in any case.

*Monoclonal antibody SK4H-12*

Table I shows the reactivity pattern of the SK4H-12 antibody (mouse immunized with SD). Positive ELISA reactions were observed with 6 of 7 TCC cell lines, 4 of which were also tested and found to be strongly stained in IFL. A strong positive fluorescence was also obtained with the bladder carcinoma of squamous cell origin (SCaBER). SK4H-12 also gave high OD values (405 nm) and an intense staining with the normal urothelial cells. With the exceptions of the prostate carcinoma line (HS), which gave a weak reaction in ELISA and the melanoma line (Ulf) giving a weak staining in IFL, no reactivity was seen with any of the controls included in this study. Furthermore, SK4H-12 gave a homogeneous staining pattern of 7/8 bladder tumour specimens but so far and as shown in Table II it did not stain any of the control tissues tested. One such reaction to a transitional cell carcinoma is illustrated in Figure 1.

**Figure 1** Transitional cell carcinoma (fresh frozen) tested in indirect immunofluorescence with antibody SK4H-12 (1:10 dilution).

*Monoclonal antibody P7A5-4*

The antibody P7A5-4 showed positive reactions with 5 out of 7 TCC cell lines, while little or no reactivity was observed with the two cells derived from normal urothelium (Table I). Furthermore, positive ELISA and/or IFL results were obtained with the squamous carcinoma line (SCaBER), 2 of 3 colon carcinomas (HT29, HCT8), the 3 melanoma cell lines (Ulf, Mel-1 and CRL-1585), a lung carcinoma cell line (A427), a prostatic carcinoma (HS) and lung fibroblasts. No reactivity was observed with the remaining controls. Results of tissue staining, summarized in Table II, show that 7 out of 8 bladder tumours tested were P7A5-4 positive. The reaction pattern was homogeneous while the intensity was moderate to strong. Among the control tissues, weak staining was associated with prostate epithelium as well as vessel endothelium in most cases. Furthermore, cryostat sections from both rat and rabbit organs (bladder, kidney, lung, liver, spleen, muscle, intestine, heart and stomach) were not stained by either of the 2 antibodies. Figure 2 illustrates the staining of a bladder carcinoma with the P7A5-4 antibody by immunoperoxidase.

*Immunoprecipitation and SDS-PAGE analysis*

To determine the cellular target structures for the 2 antibodies, precipitation was performed using NP-40 extracts of cells surface-labelled with $^{125}I$. The material bound to antibody-ProtA-Sepharose complexes was analysed on SDS-PAGE followed by autoradiography. Under reducing conditions, SK4H-12 precipitated a polypeptide with a mol. wt of $\sim$100 Kd present in extracts of SD and T24 cells (Figure 3). A similar band but at a slightly lower mol. wt was observed under non-reducing conditions. Moreover, from experiments where the extracts had been divided into a ConA binding and

**Table I** Summary of results obtained with antibodies P7A5-4 and SK4H-12 by cell-ELISA and IFL

| | | *SK4H-12* | | *P7A5-4* | |
|---|---|---|---|---|---|
| | | *ELISA* | *IFL* | *ELISA* | *IFL* |
| Transitional cell carcinoma. | TCCSuP[a] | – | – | +++ | +++(100) |
| | SD | ++ | ++(60) | – | ++ (50) |
| | EJ[a] | ++ | ++(50) | ++ | +++(95) |
| | RT4 | + | +++(90) | + | |
| | T24 | ++ | +++(50) | ++ | +++(90) |
| | Hu549 | ++ | | ++ | |
| | J82 | + | | – | |
| Squamous carcinoma. | SCaBER | | +++(60) | | +++(60) |
| Urothelial cells. | HU-609 | ++ | +++(30) | – | – |
| | HCV-29 | +++ | +++(90) | – | – |
| Colon carcinoma. | HT29 | – | – | + | |
| | HCT8 | – | – | + | |
| | LS174T | – | – | – | |
| Breast carcinoma. | MCF-7 | | – | | – |
| Malignant melanoma. | ULF | – | +(15) | + | +(15) |
| | MEL-1 | – | – | – | +(20) |
| | CRL-1585 | – | + | +[b] | |
| Osteosarcoma. | 2T | – | | – | |
| Lung carcinoma. | A427 | – | | + | |
| Prostate carcinoma. | HS | + | – | + | +(10) |
| | DU145 | –[b] | | –[b] | |
| Lung & Skin fibroblasts. | Fib | – | – | –[b] | +(50) |
| | F154 | | – | | – |
| Myeloma. | SKO | – | | – | – |
| | LICR-LON-HMy2 | – | – | – | – |
| Plasma cell leukemia. | HF-2 | –[b] | | –[b] | |
| Blood cells. | RBC ABO | – | | – | |
| | Sheep/Ox | – | | – | |
| | PBL | – | – | – | – |
| Burkitt lymphoma. | Raji | – | – | – | – |
| | Daudi | – | – | – | – |
| T cell lymphoma | Molt4 | – | – | – | – |
| Erythroleuk. | K562 | – | – | – | – |
| Histiocytic lymphoma. | U937 | – | – | – | – |
| EBV transformed lymphocytes. | EBV-lym | – | – | – | – |

*ELISA* values are given for supernatants diluted 1:100 at OD 405 nm and after 60 min. + + + = more than 1.0, + + = between 0.5 and 1,0, + = between 0.1 and 0.5, – = less than 0.1.
[a]Possible sublines of T24 (see text).
[b]Peroxidase ELISA.
*IFL* values refer to 1:10 dilutions. Figures within brackets = percentage of trained cells. + + + = strong staining of more than 50% of stained cells, + + = between 20–50% strong staining, + = less than 20% strong staining, – = no staining.

**Table II** Staining in immunofluorescence and/or immunoperoxidase by P7A5-4 and SK4H-12 antibodies (1:10 dilutions) of human tissue of different origin

| Tumour tissue | Specimens no. | Monoclonal antibodies SK4H-12 | P7A5-4[a] |
|---|---|---|---|
| Bladder carcinoma | 8 | +(7)[b] | +(7)[b] |
| Breast carcinoma | 3 | – | – |
| Pancreas carcinoma | 1 | – | – |
| Lung carcinoma | 2 | – | – |
| Colonic carcinoma | 1 | – | – |
| Prostatic carcinoma | 1 | – | +(1)[c] |
| Malignant melanoma | 5 | – | – |
| *Normal tissue* | | | |
| Bladder | 3 | — | – |
| Liver | 1 | – | – |
| Skin | 2 | – | – |
| Colon | 1 | – | – |
| Ileum | 1 | – | – |
| Tonsils | 2 | – | – |
| Thymus | 3 | – | – |
| Placenta | 2 | – | – |
| Lymph node | 1 | – | – |
| Prostate hyperplasia | 3 | – | +(2)[c] |

+ = positive reactions showing significantly elevated intensity of staining as compared to background controls. For further details see text.

Figures within brackets = number of specimens stained.

[a]Endothelium in most tissues was stained.

[b]Homogeneous reactivity pattern.

[c]Staining of epithelium lining the vesicle ducts.

— = no staining.

**Figure 2** Transitional cell carcinoma (fresh frozen) tested by indirect immunoperoxidase. (a) antibody P7A5-4 (1:10 dilution). (b) control staining with culture medium.

**Figure 3** Autoradiograph after SDS-PAGE (6–15%, reducing conditions) of different $^{125}$I-labelled NP40 extracts precipitated with P7A5-4 or SK4H-12 antibodies complexed with protein A-Sepharose 4B. TCCSuP extract precipitated with P7A5-4 antibodies (lane A) or with SK4H-12 (lane B). SK4H-12 precipitated extracts of T24 (lane C), SD (lane D), HT29 (lane E) or LS174T (lane F).

a ConA passed fraction prior to precipitation, the 100 Kd component was detected in the ConA binding fraction of a T24 cell lysate. This molecule was absent from extracts of TCCSuP, HT29 and LS174T cells (Figure 3). No precipitates were detected when the different extracts were incubated with protein A-Sepharose 4B alone.

In extracts of TCCSuP, P7A5-4 recognized 3 polypeptides (92 Kd, 23 Kd and 17 Kd) (Figure 3), a pattern that was consistent irrespective of whether the gels were run under reduced or non-reduced conditions. The two low molecular components (23 Kd and 17 Kd) were found to reside in the ConA passed fraction, while the 92 Kd was a ConA binding glycoprotein (data not shown). None of the 3 components was precipitated with lysates of SD or LS174T cells which were negative in ELISA or with HT29 which was positive.

### Discussion

The aim of this study was to raise antibodies specific for urinary bladder carcinoma and to elucidate whether these could define new structures of potential value for diagnosis and therapy. The immunizing schedule adopted was chosen to allow antigens of poor immunogenicity or low cellular expression to give rise to an immune response. For screening of hybridoma supernatants, we used IFL and a cell-ELISA similar to that described by Suter *et al.* (1980). A detailed description of the cell-ELISA developed in our laboratory has been given elsewhere (Koho *et al.*, 1984). Due to endogenous alkaline phosphatase activity or other reasons, some cell lines were tested in a peroxidase ELISA or only by IFL. Cryostat sections from frozen tissues were tested by IFL and an indirect immunoperoxidase staining method.

The occurrence of cross-contamination between urothelial cell lines has been discussed recently by O'Toole *et al.* (1983). To establish the identity of the urothelial cell lines on our target cell panel, tests for HLA A,B specificity in ADCC (O'Toole *et al.*, 1982) and HLA DR/DC specificity by DNA blotting and hybridization with cDNA probes (Andersson *et al.*, 1984), were performed (B. Karlsson *et al.*, in preparation). On the basis of these tests, 5 TCC cell lines, (T24, J82, SD, RT4, HU549) and 2 cell lines derived from normal urothelium (HU609 and HCV29) were clearly distinct from each other. When compared to T24, the 2 cell lines TCCSuP and EJ appeared to be identical in HLA A,B expression and homologous in the DR/DC loci. However, as they differed in growth pattern as well as morphology and displayed differences in antigen expression, they were included on our cell panel. The possibility that these 2 lines constitute sublines of T24 is not excluded.

When examining the antibody secreted by hybridoma SK4H-12 against a panel of cultured target cells, positive reactions were seen with 9/10 urothelium derived cells, including those of normal origin. From 25 non-urothelial cell types only 2 (HS and ULF) showed reactivity. The significance of these reactions is, however, doubtful since reactivity in both cases was only observed with one of the two assays and gave values only slightly over background. Immunohistochemical staining of tissue sections with SK4H-12 showed a similar selective pattern of reactivity. While staining the majority of TCC specimens (7/8), no reaction has so far been detected with any of the non-TCC adult tissues included in this study. Interestingly and in contrast to what was seen for cultured cells, SK4H-12 gave no visible staining of normal urothelium. Although this has to be confirmed by testing further material from normal bladder, it suggests that the cells in culture may have acquired some phenotypic characters of malignant cells. A premalignant phenotype of these cells is also supported by an apparently indefinite lifespan. However, they lack the ability to grow in nude mice and show a diploid karyotype (Vilien *et al.*, 1983).

Results of the antigen studies presented herein show that the target antigen of SK4H-12 is a glycoprotein of mol. wt 100 Kd, present in lysates of both SD and T24 cells but absent from extracts of SK4H-12 negative cell lines (TCCSuP, HT29 and LS174-T).

The other antibody, P7A5-4, displayed a specificity pattern similar to that of three other TCC-related antibodies previously found in our laboratory (Koho *et al.*, 1984). As these also precipitated polypeptides of the same molecular size, they are likely to be directed against the same target antigen. Differences in reactivity with individual cell lines suggest, however, that they recognize separate epitopes. The P7A5-4 antibody could clearly discriminate between malignant and normal cells from the bladder, both when tested with cultured cells and fresh tissue. On cell lines the antibody also showed a positive reactivity with some non-TCC and melanoma cells. However, for most of these cells the detected reactions were significantly lower indicating a quantitative difference in antigen expression by TCC and other cells. This difference in the level of expression was even more pronounced when tested with fresh tumours. A moderate to strong homogeneous staining was seen with 7 out of 8 TCC specimens while 8 non-related carcinomas as well as 5 melanomas failed to given any visible reaction. The P7A5-4 antigen was, however, not entirely restricted to TCC cells since a weak staining was also observed in association with the epithelium lining some of the vesicle ducts within the prostate as well as the vessel endothelium of some tissues. These reactivities have to be further elucidated, especially since the endothelium represented an area frequently seen to give elevated background staining. For this purpose we are presently setting up *in vitro* cultures of endothelial cells derived from the umbilical cord.

The major components precipitated with both of these antibodies, 100 Kd for SK4H-12 and 92 Kd for P7A5-4, are found in a molecular size range where TAAs of other tumours have previously been identified with mouse monoclonals. These include the 94 Kd polypeptide described by Imai *et al.* (1982) to be associated with melanomas and carcinomas and the p97 reported by two groups (Woodburry *et al.*, 1980; Dippold *et al.*, 1980) to be preferentially expressed on melanomas and some carcinomas. However, judging from the cellular distribution of these antigens as well as the lack of coprecipitated low molecular polypeptides, the 92 Kd as well as the 100 Kd appear to represent distinct antigens.

A difference in cellular restriction was also established for one of these melanoma-associated antigens, p97, when antibodies to this molecule (a gift from Dr K.E. Hellström) were tested in ELISA against the same cell panel. The two polypeptides appear also to be separate from the transferrin receptor, as antibodies to this molecule (OKT9, Sutherland *et al.*, 1981) detect a polypeptide of ~180 Kd when run on SDS-PAGE under non-reducing conditions and a band of slightly higher mol. wt (95 Kd) than the 92 Kd under reducing conditions (results not shown). Furthermore, similarity in molecular size between the major low mol. wt component (23 Kd) and the *ras* gene product p21 (Chang *et al.*, 1982) known to be well expressed in some TCC cells, raised the question whether the two molecules might be identical. However, radiolabelled TCC cell extracts precipitated by antibodies to either the p21 or the 23 Kd molecules showed distinct migration profiles (G.M. Cooper, personal communications), thus suggesting a non-identity of these molecules.

Monoclonal antibodies reactive with TCC associated antigens have recently been reported by other groups (Fradet *et al.*, 1984; Mazuko *et al.*, 1984; Messing *et al.*, 1984; Grossman, 1983). However, comparison of these findings with our own data suggests that none of these antibodies is likely to be identical to the ones described herein. Within the limits and sensitivity of the tests performed in this study, the antigenic targets for the SK4H-12 and P7A5-4 antibodies were shown to be expressed in a highly selective manner. Both were found on a majority of TCC cells but were absent from normal urothelial tissue. Although the distributional pattern of the antigens makes them potentially useful as markers for bladder carcinoma, conclusions regarding specifity have to await further tests on various tissues of malignant, normal and foetal origin.

We thank Mrs M. Karlsson for excellent technical assistance. We are also indebted to Dr K. E. Hellström (Fred Hutchinson Cancer Research Center, Seattle, Washington 98104) for providing us with anti-p97 antibodies, and to Dr G.M. Cooper (Harvard University, Sch. Med. Dana Farber Canc. Inst. Boston Ma, 02115, USA) for tests with anti-p21. This study was supported by the Swedish Cancer Society, Grant No. 365-B83-14XC.

### References

ANDERSSON, M., BÖHME, J., ANDERSON, G. & 4 others. (1984). Genomic hybridization with class II transplantation antigen cDNA probes as a complementary technique in tissue typing. *Hum. Immunol.*, **11**, 57.

CHANG, E.H., FURTH, M.E., SCOLNICK, E.M. & LOWY, D.R. (1982). Tumorigenic transformation of mammalian cells induced by a normal human gene homologous to the oncogene of Harvey murine sarcoma virus. *Nature*, **297**, 479.

CLARK, H.F. & SHEPARD, C.C. (1963). Analysis technique for preparing fluorescent antibody. *Virology*, **20**, 642.

DELAND, F.H. & GOLDENBERG, D.M. (1983). *In vivo* cancer diagnosis by radioimmunodetection. In *Radioimmunoimaging and Radioimmunotherapy*, p. 328. (Eds. Burchiel & Rhodes).

DIPPOLD, W.G., LLOYD, K.O., LI, L.T.L., OETTGEN, H.F. & OLD, L.J. (1980). Cell surface antigens of human malignant melanoma: Definition of six antigenic systems with mouse monoclonal antibodies. *Proc. Natl Acad. Sci.*, **77**, 6114.

ENGVALL, E. & PERLMANN, P. (1971). Enzyme linked immunosorbent assay (ELISA). Quantitative assay of immunoglobulin G. *Immunochemistry*, **8**, 871.

FAZEKAS, DE ST GROTH, S. & SCHEIDEGGER, D. (1980). Production of monoclonal antibodies: Strategy and tactics. *J. Immunol. Meth.*, **35**, 1.

FRADET, Y., CORDON-CARDO, C., THOMSON, T. & 5 others. (1984). Cell surface antigens of human bladder cancer defined by mouse monoclonal antibodies. *Proc. Natl Acad. Sci.*, **81**, 224.

GROSSMAN, H.B. (1983). Hybridoma antibodies reactive with human bladder carcinoma cell surface antigens. *J. Urol.*, **130**, 610.

HELLSTRÖM, K.E., HELLSTRÖM, I. & BROWN, J.P. (in press). Monoclonal antibodies to melanoma-associated antigens. In *Monoclonal Antibodies and Cancer* . (Ed. Wright Jr.) Marcell Dekker Inc: New York (in press).

IMAI, K., WILSON, B.S., BIGOTTI, A., NATALI, P.G. & FERRONE, S. (1982). A 94,000 dalton glycoprotein expressed by human melanoma and carcinoma cells. *J. Natl Cancer Inst.*, **68**, 761.

KOHO, H., PAULIE, S., BEN-AISSA, H. & 4 others. (1984). Monoclonal antibodies to antigens on transitional cell carcinoma of the human urinary bladder. I. Determination of the selectivity of 6 antibodies by cell-ELISA and immunofluorescence. *Cancer Immunol. Immunother.*, **17**, 165.

LARSON, S.M., BROWN, J.P., WRIGHT, P.W., CARRASQUILLO, J.A., HELLSTRÖM, I. & HELLSTRÖM, K.E. (1983). Imaging of melanoma with I-131-labelled monoclonal antibodies. *J. Nucl. Med.*, **24**, 123.

MACH, J.P., CHATAL, J.F., LUMBROZO, J.D. & 9 others. (1983). Tumor localization in patients by radiolabeled antibodies against colon carcinoma. *Cancer Res.*, **43**, 5593.

MAZUKO, T., YAGITA, H. & HASHIMOTO, Y. (1984). Monoclonal antibodies against cell surface antigens present on human urinary bladder cancer cells. *J. Natl Cancer Inst.*, **72**, 523.

MESSING, E.M., BUBBERGS, J.E., WHITMORE, K.E., DEKERNION, J.B., NESTOR, M.S. & FAHEY, J.L. (1984). Murine hybridoma antibodies against human transitional carcinoma-associated antigens. *J. Urol.*, **132**, 167.

NAKANE, P. & PEARCE, G.B., JR. (1966). Enzyme labelled antibodies. Preparation and application for the localization of antigen. *J. Histochem. Cytochem.*, **14**, 929.

NAYAK, S.K., O'TOOLE, C. & PRICE, Z.H. (1977). A cell line from an anaplastic transitional cell carcinoma of human urinary bladder. *Br. J. Cancer*, **35**, 142.

O'TOOLE, C.M., TIPTAFT, R.C. & STEVENS, A. (1982). HLA antigen expression on urothelial cells: Detection by antibody-dependent cell-mediated cytotoxicity. *Int. J. Cancer*, **29**, 391.

O'TOOLE, C.M., POVEY, S., HEPBURN, P. & FRANKS, L.M. (1983). Identity of some human bladder cancer cell lines. *Nature*, **301**, 429.

PAULIE, S., HANSSON, Y., LUNDBLAD, M.L. & PERLMANN, P. (1983). Lectins as probes for identification of tumor-associated antigens on urothelial and colonic carcinoma cell lines. *Int. J. Cancer*, **31**, 297.

PAULIE, S., KOHO, H., BEN-AISSA, H., HANSSON, Y., LUNDBLAD, M.L. & PERLMANN, P. (1984). Monoclonal antibodies to antigens on transitional-cell carci noma of the human urinary bladder. II. Identification of the cellular target structures by immunoprecipitation and SDS-PAGE analysis. *Cancer Immunol. Immunother.*, **17**, 173.

SHENKEIN, I., LEVY, M. & UHR, J.M. (1972). The use of glucose oxidase as generator of $H_2O_2$ in the enzymatic radioiodination of components of cell surfaces. *Cell Immunol.*, **5**, 5490.

SEARS, H.F., HERLYN, D., STEPLEWSKI, Z. & KOPROWSKI, H. (1984). Effects of monoclonal antibody immunotherapy on patients with gastroentestinal carcinoma. *J. Biol. Resp. Mod.*, **3**, 138.

SUTER, L., BRUGGEN, J. & SORG, C. (1980). Use of an enzyme linked immunosorbent assay (ELISA) for screening of hybridoma antibodies against cell surface antigens. *J. Immunol. Meth.*, **39**, 407.

SUTHERLAND, R., DELIA, K., SCHNEIDER, C., NEWMAN, R., KEMSHEAD, J. & GREAVES, M. (1981). Ubiquitous cell-surface glycoprotein on tumor cells is proliferation-associated receptor for transferrin. *Proc. Natl Acad. Sci.*, **78**, 4515.

VILIEN, M., CHRISTENSEN, B., WOLF, H. RASMUSSEN, F., HOU JENSEN, C. & POULSEN, C.O. (1983). Comparative studies of normal, spontaneously transformed and malignant human urothelium cells *in vitro*. *Eur. J. Cancer Clin. Oncol.*, **19**, 775.

WOODBURY, R.G., BROWN, J.P., YEH, M., HELLSTRÖM, I. & HELLSTRÖM, K.E. (1980). Identification of a cell surface protein, p.97, in Human melanomas and certain other neoplasms. *Proc. Natl Acad. Sci.* **77**, 2138.